# Vaccination Coverage of Greek Adults Aged ≥60 Years in a Primary Health Care Setting in Relation to Lifestyle Factors and Health Care Services Utilization

**DOI:** 10.3390/healthcare14091167

**Published:** 2026-04-27

**Authors:** Nektaria Kossyva, Marios Spanakis, Lena Borboudaki, Dimitrios Stylianakis, Nikos Rikos, Michael Rovithis, Chryssoula Perdikogianni, Manolis Linardakis, Emmanouil K. Symvoulakis

**Affiliations:** 1 Department of Social Medicine, School of Medicine, University of Crete, 70013 Heraklion, Greece; nkossyva@gmail.com (N.K.); lenaborboudaki@uoc.gr (L.B.); dimitrios.stylianakis@gmail.com (D.S.); linman@med.uoc.gr (M.L.); esymvoulakis@uoc.gr (E.K.S.); 2 Department of Nursing, School of Health Sciences, Hellenic Mediterranean University, 71410 Heraklion, Greece; 3 Department of Business Administration & Tourism, School of Management and Economics Sciences, Hellenic Mediterranean University, 71410 Heraklion, Greece; rovithis@hmu.gr; 4 Department of Pediatrics, School of Medicine, University of Crete, 70013 Heraklion, Greece; perdikogian@uoc.gr

**Keywords:** adult vaccination, Primary Health Care, healthy aging, community-dwelling elderly, lifestyle, vaccine hesitancy, behavioral health, prevention, Greece

## Abstract

**Highlights:**

**What are the main findings?**

- Influenza and COVID-19 vaccination coverage were high, whereas Tdap and RSV uptake remained low among adults aged ≥60 years.
- Higher vaccination coverage was associated with greater health care utilization, higher education level, and increased comorbidity burden.

**What are the implications of the main findings?**

- Strengthening Primary Health Care engagement and preventive service use may substantially improve adult vaccination uptake.
- Targeted educational and access-focused strategies are needed to address gaps in Tdap and RSV vaccination among older adults.

**Abstract:**

**Background/Objectives:** Vaccination represents a significant achievement of public health and should be regarded not only as a protective measure against infectious diseases but also an active preventive intervention and a component of health promotion. **Methods:** This cross-sectional study assessed vaccination coverage among adults aged ≥60 years who attended a Primary Health Care Center during a predefined period of at least two months (November–December 2025) in a rural area of Crete, Greece, and examined determinants of immunization, including demographic, clinical, psychosocial, and health service utilization factors. The sample comprised 366 participants who consented to complete a structured questionnaire, primarily via interview, followed by verification of vaccination status through medical records. **Results:** High vaccination coverage was observed for influenza (82.5%), moderate coverage for pneumococcal (68.3%) and herpes zoster (56.0%) vaccines, and very low coverage for tetanus–diphtheria–pertussis booster doses (≈13%) and RSV vaccination (5.2%). For SARS-CoV-2, 96.2% received the three doses which were mandatory during the pandemic years. The overall Vaccination Coverage Score (VCS) averaged 43.1/100, while only 10.1% of participants achieved high coverage. Regression analysis showed that higher educational level, multimorbidity, and extensive use of health services were independently associated with better vaccination coverage (*p* < 0.05). **Conclusions:** The findings reveal fragmented vaccination patterns and underscore the need for systematic assessment of adult vaccination status within routine Primary Health Care. Targeted counseling, promotion of health literacy, and preventive vaccination strategies are expected to reduce vaccine-preventable morbidity and support healthy aging.

## 1. Introduction

Vaccination represents one of the most significant achievements of public health. Technological advances have expanded vaccinology from prevention toward modulating applications and from infectious diseases to immune-mediated disorders [1]. Nevertheless, universal vaccination coverage among adults—particularly older and oldest-old populations—has not been effectively achieved and remains a major challenge for health systems and Primary Health Care (PHC) globally and nationally [2,3,4,5].

Vaccination programs have traditionally focused on infants and children whereas adult immunization has only recently become a priority [4,6,7]. One of the reasons is population aging, which represents a major societal challenge, intensifying research on older adults and healthcare systems [8,9]. Aging is characterized by the gradual accumulation of cellular damage that leads to loss of physiological integrity and decline in immune function, increasing susceptibility to infections and chronic diseases [10,11]. These processes contribute to multimorbidity and greater healthcare needs, highlighting the importance of preventive interventions [12,13]. As life expectancy rises globally, promoting healthy aging becomes essential for the sustainability of healthcare systems [14,15,16,17].

In recognition of these challenges, international recommendations and strategies have been developed to improve adult vaccination coverage. In 2009, a joint working group of the European Union Geriatric Medicine Society and the International Association of Gerontology and Geriatrics—European Region issued recommendations for vaccination in adults aged >60 years, outlining strategies to improve vaccination uptake in Europe [18,19,20]. Given that the burden of infectious diseases is greater at the extremes of age, the primary objective is healthy aging supported by lifelong immunization [7,21]. But since these initiatives, a complicating factor emerged regarding adult vaccination coverage, the COVID-19 pandemic [22,23]. Nevertheless, the Immunization Agenda 2030 (IA2030) initiative, led by international scientific bodies including the World Health Organization and UNICEF, aims to ensure that all individuals benefit from vaccination throughout the life course in conjunction with appropriate use of health services [24,25].

Vaccination protects older adults and vulnerable populations by increasing healthy life years, reducing infection transmission, lowering hospitalization rates, and mitigating the burden on healthcare systems [26,27,28]. Influenza, pneumococcal, herpes zoster, and COVID-19 vaccines are particularly important for older adults. Influenza vaccination reduces mortality, prevents co-infections, and mitigates healthcare utilization [27,29], while pneumococcal vaccination addresses a leading cause of morbidity and mortality [30,31,32]. Herpes zoster vaccination prevents painful reactivations, and COVID-19 vaccines have proven highly effective in preventing severe disease [33,34,35,36,37,38]. Other vaccines, including diphtheria, tetanus, pertussis, and respiratory syncytial virus (RSV), remain underutilized despite evidence of increased susceptibility among older adults [39,40,41,42,43].

However, despite the growing importance of adult immunization, evidence on vaccination coverage and its determinants among older adults in Primary Health Care settings remains limited, particularly in Mediterranean regions such as Greece. Vaccination should be regarded not only as a protective measure against infectious diseases but also as an active preventive intervention and a component of health promotion [17,44,45]. In rural areas with often limited healthcare facilities this can be crucial. Hence, understanding patterns of behavior—including healthcare service utilization, engagement with preventive care, and broader health-related behaviors—beyond simply recording vaccination coverage is important. Such insights can guide interventions that target behavioral health to enhance vaccination compliance, support preventive care, and ultimately improve quality of life.

Within this context, the present study aims to assess vaccination coverage for key vaccines recommended for adults aged ≥60 years who use Primary Health Care services in a rural area of Greece and to investigate associated demographic, clinical, and behavioral determinants. Findings are expected to inform targeted PHC interventions that strengthen preventive health strategies, promote active engagement in vaccination, and support healthy aging.

## 2. Materials and Methods

### 2.1. Study Design

This cross-sectional study was conducted at a Primary Health Care Center in Crete, Greece, under the supervision of the Department of Social Medicine, School of Medicine, University of Crete. The Primary Health Care Center is in the rural area of Kastelli Pediados, operating under the supervision of the 7th Health Region of Crete. The Primary Health Care Center currently actively serves a rural population of approximately 4000 residents in the district with approximately 25–30% of them being over 60 according to recent census data [46]. In this population for elderly people (≥60 years) the percentage distribution of the age groups 60–69, 70–79 and >80 is relatively homogeneous with an average percentage of around 30% for each group per area. Eligible participants were invited to enroll according to a predefined protocol including the collection of data on vaccination coverage, biometric and clinical characteristics, health behaviors, and health service utilization.

### 2.2. Ethical Approval and Informed Consent

The study protocol was approved by the 7th Health Region of Crete (Approval Number: 49520; Date: 10 November 2025). All participants were informed about the study objectives, procedures, and the voluntary nature of participation and provided written informed consent. The study was conducted in accordance with the Declaration of Helsinki and relevant national and institutional ethical guidelines. All data collected were anonymized prior to any analysis.

### 2.3. Setting and Participants

A total of *n* = 366 participants aged ≥60 years were recruited from routine outpatient visits at the Health Center during a predefined period of at least two months (November–December 2025). Participants were Greek-speaking residents of the area for at least 10 years. Exclusion criteria included cognitive or communication impairment, age < 60 years, and emergency clinical presentation. Recruitment occurred through consecutive routine appointments across three weekly clinical sessions. In total, 400 individuals were deemed eligible to participate, of whom 34 were excluded: 10 due to communication disorders and cognitive impairment, 18 due to refusal to participate or withdrawal of consent, and 6 due to acute illness. Overall, *n* = 366 participants were enrolled and provided complete data for analysis.

### 2.4. Data Collection Procedures

All interviews were conducted in a designated clinical office during routine visits. Participants were informed about the study objectives and data anonymity and provided written consent. Vaccination status was verified using medical records and used to calculate the VCS according to the national immunization program for influenza, tetanus, diphtheria, pertussis, herpes zoster, pneumococcal disease, SARS-CoV-2, and respiratory synRSV. Vaccination history was assessed in accordance with national adult immunization recommendations and standard booster schedules, in line with public health strategies aimed at improving vaccine uptake and ensuring consistent data retrieval from electronic medical records. The VCS (also expressed as a percentage) was defined based on vaccination status across these eight vaccines within the recommended timeframes. Each administered vaccine was assigned a value of 1 and non-vaccination a value of 0. If a participant has received an influenza vaccine in 2024 and 2025, they received a value of 1; if they had also received the tetanus vaccine, they also received a value of 1 and so forth with all the remaining 6 vaccines. The sum of the values received determines a score of 0–8 (0 no vaccine, 8 all vaccines), which was linearly transformed to a 0–100 scale [16]. High coverage was assessed as those with a score ≥ 66.7, a threshold that refers to the upper tertile or 2/3 of the distribution of the score 0–100.

Additional questionnaire included:

i. Sociodemographic and clinical characteristics (gender, age, subjective age perception, family status, educational level, medication use in the past 6 months, and diagnosed chronic conditions; see results Section 3.1);
ii. Health behaviors including body mass index (BMI), sleep duration, smoking, alcohol consumption, daily fruit or vegetable intake and physical activity, clustering of behavioral risk factors [47].
iii. Health Care Services Utilization score (HCSUs) based on six questions (see Results Section 3.2). The HCSUs ranged from 0 to 100 and was calculated from coded responses (0–1) to six items (sum range 0–9) followed by linear transformation [9,16]. Similarly with the VCS, high usage was assessed as those with a score ≥ 66.7, a threshold that refers to the upper tertile or 2/3 of the 0–100 score distribution.
iv. Alcohol consumption was assessed using the Greek version of the FAST-screening tool; high consumption was defined as ≥3 drinks for females and ≥4 drinks for males per occasion in the last year [47,48]. Behavioral risk factor clustering was defined as the presence of ≥3 of the following: BMI ≥ 25 kg/m^2^, smoking, high alcohol consumption, absence of daily fruit and vegetable intake (<7 days/week), and absence of daily physical activity (walking < 7 days/week) [47,48].
v. Anxiety was measured using the Greek version of the Short Anxiety Screening Test—10 (SAST-10) (score range 10–40). Categories were defined as negative (<22), borderline (22–23), and positive (≥24) [49,50].

### 2.5. Statistical Analysis

Data was analyzed using SPSS (IBM SPSS Statistics for Windows, version 25.0; IBM Corp., Armonk, NY, USA). Descriptive statistics were calculated for participant characteristics and prevalence of chronic conditions, multimorbidity, health behaviors, mental status, HCSUs, and VCSs. Percentage frequency of vaccination coverage was also reported using the corresponding 95% confidence intervals (95%CI). Cluster analysis was performed using Ward’s method and Euclidean distance for binary data for the eight vaccines, followed by dendrogram visualization. The distribution normality of HCSUs and VCs was assessed using Blom’s method (Q–Q plot). Due to slight skewness (abnormality), associations between VCs and participant characteristics, health behaviors, anxiety levels, and HCSUs were examined using Spearman’s correlation. Hierarchical multiple logistic regression analysis was also performed to assess the high VCS (≥66.7). The acceptable level of significance was set at 0.05.

## 3. Results

### 3.1. Participant Characteristics

Table 1 presents the characteristics of the 366 adults aged ≥60 years who participated in the study. Females comprised 50.8% (*n* = 186) of the sample. Participants aged ≥80 years were 30.3% (*n* = 111) of the study sample while the mean age of all was 74.6 years (SD = 8.0) and the mean subjective age was 64.1 years (SD = 19.4).

Regarding socioeconomic and health characteristics, 51.9% (*n* = 190) of participants had no or low educational attainment and 93.2% (*n* = 341) reported medication use during the previous six months. The most prevalent chronic condition was hypertension (69.9%, *n* = 242), followed by dyslipidemia (63.3%, *n* = 219) and diabetes mellitus (28.6%, *n* = 99). Multimorbidity, defined as the presence of ≥3 chronic conditions, was observed in 54.9% (*n* = 201) of participants.

### 3.2. Health Habits, Mental Status, and Health Care Services Utilization

Health-related behaviors and anxiety levels are summarized in Table 2. The mean BMI was 27.9 kg/m^2^ (SD = 4.7), with 74.3% of participants classified as overweight or obese. Participants reported a mean nightly sleep duration of 6.7 h (SD = 1.5).

Current smoking was reported by 15.9% of participants, with a mean consumption of 18.7 cigarettes per day (SD = 10.9) and a mean smoking duration of 43.3 years (SD = 11.8). High alcohol consumption during the previous year was reported by 11.2% (*n* = 41) of participants. The majority (58.5%, *n* = 214) did not consume fruits and vegetables daily, and 23.2% (*n* = 85) reported no daily physical activity. Multiple behavioral risk factors were present in 24.0% (*n* = 24) of participants.

The mean SAST score was 16.2 (SD = 4.8). Most participants were classified as negative for anxiety disorder (82.2%, *n* = 301), while 8.8% (*n* = 32) were classified as borderline and 9.0% (*n* = 33) as positive for anxiety disorder.

Health care utilization patterns are presented in Table 3. More than half of the participants (52.5%, *n* = 192) reported visiting a primary care provider up to twice annually, while 32.5% (*n* = 119) reported 3–4 visits and 15.0% (*n* = 55) reported more than four visits per year. Hospitalization within the previous three years was reported by 25.7% (*n* = 94) of participants. Nearly all participants (94.0%, *n* = 344) reported monthly out-of-pocket medication expenses, with a mean cost of €35.4.

Preventive service use was limited: 54.9% (*n* = 201) had never undergone colonoscopy screening, 10.2% (*n* = 19) of women had never undergone mammography, and 45.6% (*n* = 167) had never undergone a cardiac stress test. The mean HCSUs was 37.8 (SD = 18.0). Most participants (90.7%, *n* = 332) demonstrated low-to-moderate utilization levels, while 9.3% (*n* = 34) demonstrated high utilization.

### 3.3. Vaccination Status

Vaccination coverage levels are presented in Table 4. Influenza vaccination coverage was 82.5% (*n* = 302) for 2025 and 80.6% (*n* = 295) for 2024. Vaccination coverage for tetanus, diphtheria, and pertussis between 2017 and 2025 was low [13.1% (*n* = 48), 12.8% (*n* = 47), and 12.3% (*n* = 45), respectively]. Coverage for herpes zoster vaccination was 56.0% (*n* = 205), with 22.4% (*n* = 46) having received the second dose. Pneumococcal vaccination coverage was 68.3% (*n* = 250), and SARS-CoV-2 vaccination coverage reached 96.6% (*n* = 340) of vaccinated individuals having received three doses but none reported the updated subsequent doses. RSV vaccination coverage during 2024–2025 was 5.2% (*n* = 19).

The mean VCS was 43.1 (SD = 20.2). Most participants (89.9%, *n* = 329) demonstrated low-to-moderate vaccination coverage, whereas 10.1% (*n* = 37) achieved high coverage. Eight participants (2.2%) had received none of the assessed vaccines, whereas five participants (1.4%) had received all eight. Given that the VCS reflects the proportion of the 8 assessed vaccines received, the threshold for high coverage (VCS ≥ 66.7) corresponds approximately to receipt of at least 5–6 of the vaccines assessed in the current study.

**Figure 1:**
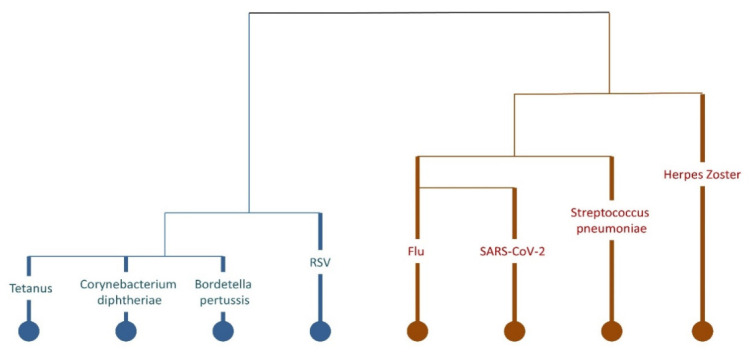
Cluster analysis dendrogram of the 8 types of vaccines on 366 participants. Colors (blue and brown) denote the two main clusters identified through hierarchical clustering, indicating vaccines with similar uptake patterns.

Hierarchical cluster analysis identified two main vaccine groupings which are combined with each other and within the groups (Figure 1):

(i) Tetanus, diphtheria, pertussis, and RSV vaccines;
(ii) Influenza, herpes zoster, pneumococcal, and SARS-CoV-2 vaccines.

### 3.4. Correlates of Vaccination Coverage and Predictors of High VCS

Associations between vaccination coverage and participant characteristics, health service utilization, health behaviors, and anxiety levels are presented in Table 5. Higher VCS values were significantly associated with male gender (rho = −0.141, *p* = 0.007), younger age (rho = −0.119, *p* = 0.023), younger subjective age perception (rho = −0.163, *p* = 0.002), higher educational level (rho = 0.203, *p* < 0.001), and multimorbidity (rho = 0.123, *p* = 0.018). Higher VCS was also associated with having undergone colonoscopy (rho = 0.133, *p* = 0.011), having undergone a cardiac stress test (rho = 0.180, *p* = 0.001), and higher HCSUs (rho = 0.129, *p* = 0.013).

Regarding health behaviors, higher VCs values were associated with daily fruit and vegetable consumption (rho = 0.104, *p* = 0.048), daily physical activity (rho = 0.120, *p* = 0.022), and lower anxiety scores (rho = −0.105, *p* = 0.045). No significant associations were observed for BMI, smoking status, alcohol consumption, sleep duration, or clustering of behavioral risk factors.

Results of hierarchical multiple logistic regression analysis are presented in Table 6. In Model 1, higher educational level (OR = 1.42, *p* = 0.008) and multimorbidity (OR = 3.52, *p* = 0.004) were independently associated with high vaccination coverage (VCS ≥ 66.7). In Model 2, with each level of increase in educational level, the odds of high vaccination coverage significantly increase (OR = 1.37, *p* = 0.022) as does the presence of multimorbidity (OR = 3.53, *p* = 0.006). Also, each unit increase in HCSUs appears to significantly increase the odds of high vaccination coverage (OR = 1.03, *p* = 0.012). Lifestyle behaviors and anxiety levels were not independently associated with VCS (*p* > 0.05).

## 4. Discussion

### 4.1. Main Findings in the Context of Existing Literature

Population aging represents a major societal challenge that has increased attention on the health needs of older adults and the sustainability of healthcare systems [8,9]. The gradual accumulation of cellular senescence due to aging mechanisms that also impact the immune system increases the vulnerability to infections and contributes to multimorbidity [10,11,12]. Promoting healthy aging becomes increasingly important, and vaccination constitutes a key preventive intervention for reducing the burden of vaccine-preventable diseases and their complications among older adults [6,14,20]. Vaccination should be seen as a prospective measure for an individual to achieve healthy aging [51]. Therefore, addressing existing gaps and barriers that hinder the advancement of a life course approach to vaccination is essential to guide approaches for strengthening vaccination compliance [14,17,35,44,52,53].

The present study evaluated vaccination coverage among individuals aged over 60 years according to the Greek national adult vaccination program. The study population was characterized by a considerable clinical burden, with a high prevalence of multimorbidity and frequent pharmaceutical treatment, suggesting a population that could benefit substantially from preventive measures. Despite this profile, overall vaccination coverage remained moderate, with only a small proportion of participants achieving high vaccination coverage. This finding suggests that vaccination among older adults is often implemented selectively rather than as part of a comprehensive preventive strategy. All assessed vaccines are provided free of charge to eligible adults under the national immunization program in Greece. Therefore, financial barriers were unlikely to significantly influence vaccination uptake in our study population.

Influenza vaccination coverage was particularly high, exceeding 80%, a finding consistent with previous studies in Greece [33,42,54]. This may reflect the long-standing implementation of national vaccination campaigns and the active involvement of community pharmacies in vaccine administration [33,55,56]. Influenza infection is associated with increased healthcare utilization, complications, and hospitalizations among older adults, highlighting the importance of vaccination in reducing disease burden and healthcare system pressure [57,58,59,60,61,62,63].

SARS-CoV-2 vaccination coverage was deemed high regarding the mandatory doses, with 96.6% of the sample having received a third dose. As for later updated doses, negligence and aversion to continuing receiving it was reported. Widespread use of COVID-19 vaccines was necessary for controlling the COVID-19 pandemic. Randomized trials for COVID-19 vaccines demonstrated approximately 95% efficacy and protection against severe disease [36,37,38]. Among the elderly, the benefits of vaccination far outweigh potential risks [64]. Generally, the results of this work are comparable with previously published data as to the vaccine coverage for SARS-CoV-2, the mandatory three dosages and the subsequent updated ones [54,65,66,67]. The annual immunization for high-risk groups indicates the need of a stable acceptance of the vaccine in the elderly despite any vaccination fatigue. One strategy to enhance uptake is to promote the fact that COVID-19 vaccination is part of a routine, annual immunization schedule for vulnerable groups—like influenza vaccines—rather than a single, isolated dose. Cultivating this understanding is critical, as it may positively influence vaccination compliance and support broader efforts to increase coverage in the community [24,68,69,70,71].

In contrast, vaccination coverage for diphtheria, tetanus, and pertussis was low. Previous studies have shown that protective antibody levels for these infections decline with age, increasing vulnerability among older populations [39,40,41,42]. Similarly, tetanus cases are disproportionately reported among individuals over 65 years of age, while pertussis has also been associated with increased mortality in older adults [72,73,74].

Pneumococcal vaccination coverage in the present study was relatively high compared with previous Greek studies [33,42,54]. Considering that respiratory infections remain a major cause of morbidity and mortality worldwide, pneumococcal vaccination represents an important preventive intervention for older adults [31,32,75,76]. Herpes zoster vaccination coverage was also higher than previously reported in Greek populations, which may reflect increased preventive awareness or local characteristics of the healthcare system and population [33,34,77].

Conversely, vaccination coverage for RSV was very low, likely due to the recent introduction of this vaccine into adult immunization programs and limited public awareness [43,78]. Nevertheless, RSV is increasingly recognized as an important cause of respiratory infection among older adults and is associated with significant morbidity, hospitalization, and mortality [79,80,81,82]. Emerging evidence indicates substantial vaccine effectiveness in preventing severe outcomes [83,84,85].

Overall, the clustering of influenza and COVID-19 vaccination coverage and the subsequent findings regarding the rest of the vaccine compliance as observed in this study suggests that vaccination behavior may be influenced by perceived disease risk and the intensity of public health communication [68,77,86,87]. Vaccines associated with widely discussed or urgent health threats appear to achieve higher coverage, whereas vaccines linked to long-term complications or less visible diseases may receive lower priority [69,88,89].

Several demographic and clinical factors were associated with vaccination coverage. Gender differences were observed, with men demonstrating higher vaccination rates than women, a pattern previously reported in other studies for influenza and COVID-19 vaccines [90,91,92,93]. Educational level was positively associated with vaccination coverage, supporting the importance of health literacy in preventive decision-making [94,95,96]. Multimorbidity was also associated with higher vaccination coverage, possibly because individuals with multiple chronic conditions have more frequent interactions with healthcare services and greater perceived vulnerability to infectious diseases [54,97,98].

Healthcare service utilization was generally low in the study population, although higher utilization was associated with higher vaccination coverage. Preventive examinations such as colonoscopy and cardiac stress testing were also linked to higher vaccination rates, suggesting that vaccination may be part of a broader pattern of preventive healthcare behaviors. These findings highlight the important role of Primary Health Care in promoting vaccination among older adults, as regular contact with healthcare providers creates opportunities for vaccination recommendations and patient education [71,86,99,100].

Vaccination coverage among older adults has important implications for healthcare system sustainability and population health outcomes. As aging populations increase the burden of vaccine-preventable diseases, vaccination remains a cost-effective strategy to reduce complications, healthcare utilization, and mortality [4,17,20,28]. However, vaccination coverage often remains suboptimal despite free vaccination programs, indicating that psychological, educational, and social barriers also influence vaccine uptake [7,28,88,90,101]. Factors such as vaccine confidence, perceived risk, digital literacy, and sociocultural influences may shape vaccination decisions [26,27,102,103,104,105]. Strengthening vaccination strategies within Primary Health Care and promoting a life course approach to immunization may therefore contribute to improving vaccination coverage and supporting healthy aging.

### 4.2. Strengths, Limitations, and Directions for Future Research

This study provides a comprehensive assessment of vaccination coverage across multiple vaccines in a community-dwelling older population, while simultaneously examining sociodemographic, behavioral, psychological, and health system factors. The inclusion of a composite VCS and health care utilization index strengthens the analytical framework.

However, several limitations should be acknowledged. The cross-sectional design does not permit causal inference. Self-reported data may introduce recall bias. The use of factors, mainly personal characteristics, was assessed as marginally non-significant in their relationship with vaccination coverage, while overall a low coefficient of determination (R^2^) was attributed to the parameters evaluated. The study population also represents a specific geographic region, which may limit generalizability. Also, a potential selection bias related to recruitment during routine outpatient visits should be considered, as individuals with higher healthcare utilization may be more likely to be included. However, the study was conducted in the only major Primary Health Care facility in the area, which serves the entire local population through both regular and unscheduled visits, thereby reducing, but not eliminating, the risk of systematic selection bias. In addition, although the mean VCS suggests moderate coverage, its skewed distribution indicates substantial heterogeneity, with few participants at the extremes. Under the current protocol, this pattern was not explored in depth and warrants further investigation, ideally in larger cohorts to better understand selective vaccination behaviors.

Future research should move beyond cross-sectional designs by adopting longitudinal and mixed methods approaches to better capture changes in vaccination behavior over time. The use of objective data sources, such as electronic health records or immunization registries, would improve validity and reduce recall bias. Studies should also identify distinct vaccination profiles and test targeted interventions, including reminder systems and primary care–based or pharmacist-led strategies. Greater emphasis on psychosocial determinants—such as risk perception, vaccine confidence, and cognitive biases—may further clarify drivers of vaccine uptake, particularly among vulnerable subgroups, including the oldest-old, socially isolated individuals, and those with low health literacy.

### 4.3. Implications for Practice and Policy

Findings highlight the importance of strengthening Primary Health Care engagement, promoting participation in preventive screenings, and implementing targeted communication strategies [47,106,107]. Policies should prioritize systematic vaccination reminders for older adults, the integration of vaccination with chronic disease management, targeted outreach to socially isolated individuals, expansion of health literacy programs, and improved accessibility and convenience of vaccination services. Community pharmacists, who actively perform adult vaccinations such as influenza, represent an essential resource for increasing vaccination coverage, improving adherence, and enhancing preventive care in older adults [56,108]. Life course immunization strategies should be embedded within broader healthy aging policies to ensure sustained protection against vaccine-preventable diseases and to support the overall health and well-being of the aging population [109]. Educational strategies should extend beyond information provision to address misconceptions, fear, and cognitive biases. Techniques derived from cognitive-behavioral approaches may help reduce overestimation of vaccine-related risks and improve decision-making [110]. Tailored interventions could be particularly relevant for individuals with low health literacy or high uncertainty. A unified prevention concept may overcome health decision fragmentation or service inertia in the future.

## 5. Conclusions

Vaccination coverage among adults aged ≥60 years in Greece is high for influenza, moderate for pneumococcal and herpes zoster, and low for diphtheria-tetanus-pertussis and RSV. Factors associated with higher coverage include male sex, higher educational level, multimorbidity, preventive health behaviors, and greater use of healthcare services. Increasing age was associated with lower vaccination uptake in this study, although the underlying mechanisms were not directly assessed. These findings underscore the importance of life course vaccination strategies, targeted education, and accessibility improvements, particularly for adults with low healthcare utilization or limited preventive care engagement. Strengthening adult vaccination in primary care can improve healthy aging, reduce infectious disease burden, and enhance the sustainability of healthcare systems.

## Figures and Tables

**Table 1:** Demographic and health characteristics of 366 participants (60+ years) in the study.

		*n*	%
**Gender**	males/females	180/186	49.2/50.8
**Age**, (years)	mean ± stand. dev. (median, IQR)	74.6 ± 8.0 (74.5, 14.4)	
	60–69	125	34.2
	70–79	130	35.5
	≥80	111	30.3
**Subjective sense of Age**, (years)	mean ± stand. dev. (median, IQR)	64.1 ± 19.4 (65.0, 30.0)	
**Family status**	married, in relationship	264	72.1
	unmarried, divorced, widows	102	27.9
**Educational level**	minimum/no education	190	51.9
	Secondary	55	15.0
	High school	54	14.8
	Technical School	34	9.3
	University	33	9.0
**Taking medication during the last 6 months**	yes	341	93.2
**Chronic conditions** (most frequent) ^a^	hypertension	242	69.9
	dyslipidemia	219	63.3
	diabetes mellitus	99	28.6
	thyroid disease	81	23.4
	coronary heart disease	74	21.4
**Multimorbidity**	≥3 chronic conditions	201	54.9

IQR, interquartile range; ^a^ 21 chronic conditions such as hypertension, dyslipidemia, diabetes mellitus (I, II), thyroid disease, coronary heart disease, osteoarthritis, chronic obstructive pulmonary disease, atrial fibrillation, osteoporosis, heart failure, rheumatoid arthritis, cancer, allergy, asthma, chronic kidney failure, acute myocardial infarction, stroke, dementia, peripheral angiopathy, hepatitis and Parkinson’s disease.

**Table 2:** Health habits and anxiety levels of 366 participants in the study.

			*n*	%
**Body Mass Index**, (kg/m^2^)		mean ± stand. dev. (median, IQR)	27.9 ± 4.7 (27.7, 5.6)	
		underweight (<18.5)	5	1.4
		normal (18.5–24.9)	89	24.3
		Overweight, obese (≥25.0)	272	74.3
**Night-time sleep hours**		mean ± stand. dev. (median, IQR)	6.7 ± 1.5 (7.0, 2.0)	
**Smoking**		currently smoking	58	15.9
		non-smoker	186	50.8
		ex-smoker	122	33.3
	cigarettes/day	mean ± stand. dev. (median, IQR)	18.7 ± 10.9 (20.0, 10.0)	
	years of smoking	mean ± stand. dev. (median, IQR)	43.3 ± 11.8 (45.0, 10.0)	
**High alcohol consumption** [≥3 drinks (♀) or ≥4 (♂) per occasion during the last year or FAST score ≥ 3]			41	11.2
**Daily consumption of fruits & vegetables**		no	214	58.5
		yes	152	41.5
**Daily physical activity** (walking for ≥10 min/day)		no	85	23.2
		yes	281	76.8
**Multiple behavioral risk factors** (MBRFs) ^a^		0–2	278	76.0
		≥3 or multiple presence	24	24.0
**Short Anxiety Screening Test—SAST scale** ^b^		mean ± stand. dev. (median, IQR)	16.2 ± 4.8 (15.0, 7.0)	
		negative for anxiety disorder (<22)	301	82.2
		borderline (22–23)	32	8.8
		positive (≥24)	33	9.0

IQR, interquartile range; ^a^ behavioral risk factors for chronic diseases are referred to the unhealthy habits as: high body weight (BMI ≥ 25.0 kg/m^2^), smoking habit, alcohol consumption (FAST score ≥3/4 per gender), absence of daily consumption of fruits and vegetables (<7 days/week) and absence of daily physical activity (walking < 7 days/week). The clustering of multiple behavioral risk factors is based on the multiple presence of 3 or more factors. ^b^ Score was extracted by summing up the responses of 10 items, ranging from 10 to 40. The higher the score, the higher the anxiety.

**Table 3:** Components (questions) of HCSUs in participants in the study.

Relevant Questions	Scoring	*n*	%
How often do you visit a health center, regional clinic, or private clinic for medical reasons within a year?	0: 0–2 times	192	52.5
	1: 3–4	119	32.5
	2: >4	55	15.0
Have you been hospitalized in the last 3 years?	0: never	272	74.3
	1: 1 time	67	18.3
	2: 2 times	14	3.8
	3: 3 or more	13	3.6
How much do you spend on co-payments for medications each month?	mean ± stand. dev. (median, IQR)	35.4 ± 26.5 (30.0, 30.0)	
	0: 0 euros	22	6.0
	1: at least 1 euro	344	94.0
Have you ever had a preventive colonoscopy?	0: no	201	54.9
	1: yes	165	45.1
Have you ever had a preventive mammogram? (♀, n = 186)	0: no	19	10.2
	1: yes	167	89.8
Have you ever taken a cardio-stress test?	0: no	167	45.6
	1: yes	199	54.4
**Health Care Services Utilization score** (HCSUs)	mean ± stand. dev. (median, IQR)	37.8 ± 18.0 (33.3, 22.2)	
	low to moderate (0–66.6 or 2/3)	332	90.7
	high (≥66.7)	34	9.3

IQR, interquartile range; score ranges from 0 to 100, with a higher score indicating greater use of health services. The assessment was based on the coded responses (scoring 0 and 1) to the six questions. After they were added up with a possible range of 0–9, a linear transformation was performed on a scale of 0–100.

**Table 4:** Frequency of VCs and assessment of relative score in 366 adults aged 60+ years, participants in the current study.

Types of Vaccines	Year and Doses of Vaccination	*n*	%	95%CIs
Influenza	2025	302	82.5	78.4, 86.1
	2024	295	80.6	76.3, 84.4
	2024 & 2025 (1) ^a^	295	80.6	76.3, 84.4
Tetanus	from 2017 to 2025 (1)	48	13.1	10.0, 16.9
Corynebacterium diphtheriae	from 2017 to 2025 (1)	47	12.8	9.7, 16.6
Bordetella pertussis	from 2017 to 2025 (1)	45	12.3	9.2, 16.0
Herpes Zoster	from 2017 to 2025 (1)	205	56.0	50.9, 61.0
	1 dose	159	77.6	71.5, 82.9
	2 doses	46	22.4	17.1, 28.5
Streptococcus pneumoniae	from 2014 to 2025 (1)	250	68.3	63.4, 72.9
SARS-CoV-2	from 2021 to 2025 (1)	352	96.2	93.8, 97.8
	1 dose	1	0.3	
	2 doses	11	3.1	
	3 doses	340	96.6	
RSV (Respiratory Syncytial Virus)	2024 & 2025 (1)	19	5.2	3.3, 7.8
**Vaccination Coverage Score (VCS)**	mean ± stand. dev. (median, IQR)	43.1 ± 20.2 (50.0, 25.0)		
	low-to-moderate (0–66.6 or 2/3)	329	89.9	86.5, 92.7
	high (≥66.7)	37	10.1	7.3, 16.5

IQR, interquartile range; the VCS (also expressed as a percentage) was defined based on the administration of the above eight vaccines, for the periods of time, either in combination or individually, to which they relate. For each vaccine administered, a value of 1 was given (^a^ respectively in parentheses) and for non-vaccination a value of 0. After adding them up with a possible range of 0–8, a linear transformation was performed on a scale of 0–100. It is noticed that without any vaccine were found *n* = 8 participants (2.2%) as with eight vaccines were found *n* = 5 participants (1.4%).

**Table 5:** VCS for 366 participants as to their demographic characteristics, the components (questions) HCSUs, health habits and anxiety levels.

		VCS (0–100)	
		Rho-Spearman	*p*-Value
**Demographic** **Characteristics**	**Gender** (1: males, 2: females)	−0.141	0.007
	**Age** (1: 60–69 years, 2: 70–79, 3: ≥80)	−0.119	0.023
	**Subjective sense of age** (years)	−0.163	0.002
	**Family status** (1: married, in relationship, 2: unmarried, divorced, widows)	−0.168	0.001
	**Educational level** (1: minimum/no education, 2: Secondary, 3: High school, 4: Technical School, 5: University)	0.203	<0.001
	**Taking medication during the last 6 months** (1: no, 2: yes)	0.019	0.718
	**Multimorbidity** (1: 0–2 chronic conditions, 2: ≥3)	0.123	0.018
**Components (questions) &** **HCSUs**	*How often do you visit a health center, regional clinic, or private clinic for medical reasons within a year?* (0: 0–2 times, 1: 3–4, 2: >4)	0.085	0.105
	*Have you been hospitalized in the last 3 years?* (0: never, 1: 1 time, 2: 2 times, 3: 3 or more)	0.060	0.122
	*How much do you spend on co-payments for medications each month?* (euros)	0.009	0.288
	*Have you ever had a preventive colonoscopy?* (0: no, 1: yes)	0.133	0.011
	*Have you ever had a preventive mammogram?* (♀, *n* = 186) (0: no, 1: yes)	0.009	0.900
	*Have you ever taken a cardio-stress test?* (0: no, 1: yes)	0.180	0.001
	**Health Care Services Utilization score—HCSUs** (0–100)	0.129	0.013
**Health habits and anxiety levels**	**Body Mass Index** (kg/m^2^)	0.070	0.200
	**Night-time sleep hours**	0.008	0.639
	**Smoking** (1: non, ex-smokers, 2: currently smokers)	0.028	0.591
	**High alcohol consumption** (FAST score as 1: up to 2 & 2: ≥3)	−0.033	0.530
	**Daily consumption of fruits & vegetables** (1: no, 2: yes)	0.104	0.048
	**Daily physical activity** (1: inactivity or walking for less than 10 min/day, 2: walking for ≥10 min/day)	0.120	0.022
	**Multiple behavioral risk factors (MBRFs)** (with none up to 5)	−0.057	0.303
	**Short Anxiety Screening Test—SAST scale** (score)	−0.105	0.045

**Table 6:** Hierarchical multiple logistic regression analysis of 366 participants with high VCS (≥66.7) in relation to those with low-to-moderate and their characteristics, the HCSUs, the health habits and anxiety levels.

	VCS (*High* Versus *Low-to-Moderate*)			
	1st Model		2nd Model	
*Prognostic Factors*	Odds Ratio (95%CI)	*p*-Value	Odds Ratio (95%CI)	*p*-Value
**Gender** (females vs. males)	0.48 (0.20, 1.06)	0.067	0.47 (0.19, 1.03)	0.080
**Age** (per decade change or 1: 60–69 yrs, 2: 70–79 and 3: ≥80)	0.75 (0.43, 1.23)	0.289	0.75 (0.43, 1.31)	0.319
**Family status** (unmarried, divorced or widows vs. married or in relationship)	0.23 (0.05, 1.01)	0.052	0.25 (0.06, 1.17)	0.073
**Educational level** (per level change or 1: minimum/no education, 2: Secondary, 3: High school, 4: Technical School, 5: University)	1.42 (1.15, 1.93)	0.008	1.37 (1.10, 1.89)	0.022
**Multimorbidity** (≥3 chronic conditions ***vs.*** 0–2)	3.52 (1.77, 9.30)	0.004	3.53 (1.52, 8.78)	0.006
**Health Care Services Utilization score—HCSUs** (per unit change in the scale of 0–100)			1.03 (1.01, 1.04)	0.012
**Daily consumption of fruits & vegetables** (yes ***versus*** no)			1.90 (0.93, 4.22)	0.098
**Daily physical activity** (walking for ≥10 min/day ***versus*** inactivity or walking for less than 10 min/day)			2.24 (0.66, 8.68)	0.224
**Short Anxiety Screening Test—SAST scale** (per unit change in the scale of 1–40)			0.96 (0.31, 1.69)	0.392
R^2^ Nagelkerke	0.20		0.26	

## Data Availability

The data presented in this study are available on request from the corresponding authors due to confidentiality requirements related to the use of individual-level medical records and electronic health data.
